# Frozen embryo transfer prevents the detrimental effect of high estrogen on endometrium receptivity

**DOI:** 10.4274/jtgga.2016.0186

**Published:** 2017-03-01

**Authors:** Aynur Adeviye Erşahin, Mustafa Acet, Suat Süphan Erşahin, Nur Dokuzeylül Güngör

**Affiliations:** 1 Department of Obstetrics and Gynecology, Bahçesehir University Faculty of Medicine, İstanbul, Turkey; 2 Department of Obstetrics and Gynecology, Medipol University Faculty of Medicine, İstanbul, Turkey; 3 Department of Obstetrics and Gynecology, Kemerburgaz University Faculty of Medicine, İstanbul, Turkey; 4 Department of Obstetrics and Gynecology, Medicalpark Göztepe Hospital, İstanbul, Turkey

**Keywords:** Frozen embryo transfer, fresh embryo transfer, clinical pregnancy rates, serum estradiol, serum progesterone

## Abstract

**Objective:**

To investigate whether serum levels of estradiol affect reproductive outcomes of normoresponder women undergoing fresh embryo transfer (ET) versus frozen-thawed ET (FET).

**Material and Methods:**

Two hundred fifty-five normoresponder women underwent fresh ET in their first or second in vitro fertilization cycle. Ninety-two women with negative pregnacy test results underwent FET. Clinical and ongoing pregnancy rates, implantation, and live birth rates of women undergoing fresh ET versus FET were compared.

**Results:**

One hundred forty-seven (57.65%) out of the 255 normoresponder women receiving FET had positive beta-human chorionic gonadotrophin (hCG) results. The remaining 108 women had negative beta-hCG results. The clinical pregnancy rates of the fresh ET group were found as 55.69% (n=142). Ninety-two of the 108 women with failed pregnancies underwent FET; 72.83% had positive beta-hCG results (n=67), and 70.65% had clinical pregnancy (n=65). Both biochemical and clinical pregnancy rates of women undergoing FET increased significantly (p<0.012 and p<0.013, respectively). Ongoing pregnancy (60.87% vs. 52.94%) and live birth rates (59.87% vs. 48.63%) were similar in both fresh and FET groups. Serum E2 levels of women who failed to conceive were significantly higher than those women did conceive. Serum progesterone levels of women who conceived versus those that did not were similar.

**Conclusion:**

The detrimental effect of high serum estradiol levels on endometrial receptivity could be prevented by FET.

## INTRODUCTION

High estradiol levels on the day of human chorionic gonadotrophin administration were found to be detrimental upon endometrial receptivity in women undergoing *in vitro* fertilization (IVF)/intracytoplasmic sperm injection (ICSI) (1). By altering serum levels of estrogen and progesterone, controlled ovarian stimulation (COS) with recombinant or urinary follicle-stimulating hormone (FSH) might alter endometrial receptivity either positively or negatively (2, 3). Fluctuation in serum estradiol or progesterone levels might lead to asynchrony between embryo and endometrium. It is well known that synchrony between the implanted blastocyst and endometrium increases implantation rates. On the other hand, asynchrony between the transferred embryo and endometrium may lead to implantation failures, despite the transfer of sufficient numbers and good quality embryos (4, 5, 6, 7, 8).

Fresh embryo transfer (ET) and frozen-thawed (FET) are the most common ET methods. Implantation rates of frozen-thawed embryos reached the success rates of fresh embryos with the development of vitrification technologies (9, 10). In particular, infertile women with slowly developing embryos or premature progesterone peaks might benefit more from FET than with fresh ET. Endometrial priming in frozen ET with the use of exogenous hormones may lead to strict control of endometrial development. In FET cycles, the endometrium is artificially primed with E2 and progesterone and embryos are therefore transferred to an environment that has not been exposed to the effects of high estradiol and progesterone levels that occur during COS. Previous studies used different infertile participants to compare the impact of fresh and frozen ET on reproductive outcomes (11). Unlike others, we examined the clinical outcomes of a cohort of young patients underdoing fresh ET, and subsequently compared the outcomes of a subset of these patients who failed with fresh transfer and subsequently underwent frozen single ET. Thus, we will have the opportunity to more objectively analyze the influences of COS-related hormonal alterations on endometrium receptivity.

## MATERIAL AND METHODS

### Patient selection

This study included 255 patients who received fresh ET in their first or second treatment cycle. An increase in serum levels of human chorionic gonadotrophin (hCG) within 10-12 days after fresh or frozen ET was accepted as pregnancy. One hundred eight of 255 participants had negative beta-hCG results, 92 of whom underwent frozen ET. The clinical and ongoing pregnancy rates, implantation, and live birth rates of women undergoing fresh and frozen ET were compared. Ongoing pregnancy was accepted as the main outcome measure. Detection of fetal heart motion at 6-7 weeks’ gestation was noted as clinical pregnancy. Fetuses with fetal heart motion at 12 weeks’ gestation were accepted as ongoing pregnancy. The implantation rate was defined as the ratio of the number of transferred fresh or frozen embryos that resulted in fetal heart activity. Pregnancy losses within the early gestational period was pregnancy but it did not become ongoing pregnancy. Institutional review board approval was obtained before initiation of this retrospective study. The inclusion criteria were i) patients undergoing their first or second IVF cycle; ii) cycle day 3 FSH <10 IU/L; and iii) 10-15 antral follicles observed on baseline ultrasonography; iv) 10-15 oocyte collection at oocyte pick-up. Patients with a history of recurrent implantation failure, recurrent spontaneous abortions, poor responders, and high responders were excluded. The mean age of participants was less than 35 years.

### Controlled ovarian stimulation protocol

The protocols for COS, embryo culture, cryopreservation, and luteal support were described previously. In brief, patients underwent COS with recombinant FSH (rFSH; Gonal-F; Merck Serono, Turkey) and gonadotrophin-releasing hormone (GnRH) antagonist (Cetrotide; Merck Serono, Turkey). When the leading follicle reached a diameter of 12-13 mm, the GnRH antagonist was administered 0.25 mg daily until the hCG injection. When two or more follicles had attained a minimum mean diameter of 18 mm, follicular maturation was achieved using 250 μg of r-hCG (Ovitrelle; Merck Serono, Switzerland). Oocyte retrieval was performed 36 h after the hCG injection. Luteal phase support was given with vaginal progesterone gel until the detection of the fetal heartbeat.

### Endometrial priming for frozen embryo transfer

Participants in the frozen ET group underwent endometrial priming with oral 6.0 mg/daily estradiol (Estrofem; Novo Nordisk, Denmark). It began on day 3 of menses and continued for 10-14 days. E2 patch supplementation was used if needed. Priming was continued until the endometrial thickness reached at least 8 mm. Luteal phase support was given to both the fresh and frozen ET group with vaginal P gel (Crinone; Merck Serono, Turkey). P gel was used twice a day beginning from five days before the thawing and continued until week 12.

### Fresh and frozen embryo selection

Embryo morphology was assessed according to the number, symmetry, percentage of fragmentation, presence of multinucleated blastomeres, and degree of compaction (12). Blastocysts were scored according to Gardner’s classification. Blastocysts with best-morphology were selected for fresh ET. Grade 3AA and above blastocysts were vitrified in turn on a cryotop (Kitazato; Japan) using a commercially available kit (Vitrolife; Sweden). Blastocysts were also thawed using the same kit following the manufacturer’s instructions.

### Statistical analysis

Data analysis was performed using the Statistical Package for Social Sciences version 15.0 (SPSS Inc, Chicago, IL, USA). Normality of distributions were checked with the Kolmogorov-Smirnov test. Statistical differences in continuous variables were determined using Student's t-test and the Mann-Whitney U test, if appropriate. Chi-squared and Fisher’s exact tests were used to analyze categorical data. P<0.05 was considered statistically significant.

## RESULTS

All participants had good ovarian response, which allowed the retrieval of 13 cumulus oocyte complexes and 10 metaphase II oocytes. The age and body mass index (BMI) of the participants were found to be 27.9±3.72 years and 24.7±3.24 (kg/m^2^), respectively. All fresh cycles were transferred on the same day. One hundred forty-seven (57.65%) of the 255 normoresponder women who received FET had positive beta-hCG results. The remaining 108 women had negative beta-hCG results. The clinical pregnancy rate was found as 55.69% (n=142). A subgroup analysis of patients who failed fresh transfer and subsequently underwent FET demonstrated that there was no demographic differences between the two groups. However, a cycle-based difference was detected regarding serum estradiol levels on hCG day between patients undergoing fresh transfers who conceived versus those that did not.

Serum E2 levels of women who failed to conceive were significantly higher than those who conceived succesfully (p<0.01; Table 1). Nevertheless, similar serum progesterone levels were noted in women who conceived versus those that did not (p>0.65). The time between fresh and frozen transfers were at least one year. The maturation and fertilization rates of fresh oocytes were found as 83.50% and 87.02%, respectively. The transferred blastocysts were of top quality for fresh cycles (55% vs. 27%) and of good quality for frozen-thawed cycles (72.8% vs. 27%). Ninety-two of 108 women who failed pregnancy underwent FET, 72.83% (n=67) of whom had positive beta-hCG results, and the clinical pregnancy rate was 70.65% (n=65). Both biochemical and clinical pregnancy rates of women undergoing FET increased significantly (p<0.012 and p<0.013, respectively). Ongoing pregnancy (60.87% vs. 52.94%) and live birth rates (59.87% vs. 48.63%) were similar in both fresh and frozen ET groups. Finally, the cumulative clinical pregnancy rate was 81.17%. Clinical miscarriage rates of women undergoing forzen ET cycles were significantly higher than those in fresh ET cycles (p<0.045). However, early pregnancy loss in the fresh ET group was significantly higher than in the frozen ET group (p<0.03).

## DISCUSSION

Great efforts have been made over the last two decades to improve clinical and embryologic strategies with the aim of improving outcomes of assisted reproductive technologies. The endometrium is accepted as a final destination allowing blastocysts to attach under sufficient amounts of biologically-relevant receptivity molecules. Understanding endometrial receptivity, or more accurately, detecting the window of implantation, has become crucial in ART practise in order to go one step further (13, 14). In line with this, improvements in blastocyst culture medium combined with robust development in vitrification protocols have undeniably improved the impact of COS cycles. Accorrdingly, two large retrospective studies reported that FET cycles had equivalent reproductive outcomes to fresh cycles (15, 16). Despite the positive impact of COS on the number of oocytes collected, COS may lead to defective endometrial receptivity. Supraphysiological estrogen production may be the main culprit responsible for the failed receptivity in women having high estradiol levels (1). Concordantly, it has been reported that high serum estradiol levels on the day of HCG stimulation in women who are high or normal responder are detrimental to endometrial receptivity (17). Therefore, we conducted a retrospective cohort study with the aim of comparing reproductive outcomes of fresh ET versus FET in the same cohort of 255 young patients who were normoresponders in order to assess possible impact of COS cycles on endometrial receptivity.

As clinical pregnancy rates were higher in the subset of patients undergoing frozen transfer as opposed to the entire first cohort undergoing fresh transfer, we conclude that fresh cycles are hindered by impaired endometrial receptivity. However, de Neubourg et al. (18) reported a 5% increase in cumulative live birth rate as an additional effect of frozen-thawed cycles. Conversely, in the present study, the cumulative increase in pregnancy rates was about 25%. The difference betwen the two studies could be accounted for by the fact that our study used blastocyst vitrification, whereas de Neubourg et al. (18) used a slow freezing protocol on day 3 embryos. There may be different reasons for the increase in success rates of frozen ET. First, endometrial priming with estradiol and progesterone provide a natural endometrial environment for the transferring embryo in frozen ET cycles. Secondly, detrimental impacts of COS-related hormonal fluctuations on endometrium receptivity can be prevented by using frozen ET cycles, in which ovarian stimulation with rFSH or urinary FSH are not used. Moreover, disturbed expression of the receptivity genes and molecules in the endometrium during the window of implantation might be a common factor among patients undergoing COS due to different etiologies. In good agreement, failed endometrial receptivity has been noted in some COS cycles with high serum estradiol levels (1). Treatment of rats with 100 ng estradiol per day on gestation days 1-5 lead to complete absence of implantation sites, which supports the detrimental effect of high estrogen levels on implantation site (19). Likewise, we found that serum estradiol levels of women who did not conceive were significantly higher than those of women who conceived successfully.

Sevaral mechanisms may be responsible for increased pregnancy rates after FET cycles. Similar ongoing and live birth rates in both transfer groups suggest that COS per se, or COS-related defects alone do not disturb the expression of endometrial receptivity molecules. However, we do not know whether the increase in clinical pregnancy rates after FET cycles is associated with removal of high E2 levels or a consequence of other factors associated with the underlying disease. Down-regulation of serum E2 levels could be a direct cause of increased pregnacy rates. FET-related improvement in COS-induced hormonal fluctuations including high E2 and proesterone levels may lead to an increase in clinical pregnancy rates. To avoid COS-related detrimental effects on endometrium receptivity in the present study, all FET cycles were made at least one year after the fresh ET.

In the present study, despite higher biochemical and clinical pregnancy rates in FET cycles possible explanation of equivalent live birth and higher miscarriage rates in both groups of subjects are unclear. We can propose that although frozen transfers may indeed benefit some patients who with impaired endometrial receptivity after COS, we have to select our results more carefully to discern whether there is a subset of patients at highest risk for fresh transfer failure (i.e., potential patients with higher E2 or progesterone levels). E2-stimulated endometrium may be the source of implantation failure in some fresh IVF cycles; however, this may not be the only reason (20, 21). If so, abortion rates and live birth rates were not the same in both groups. If we proclaim high serum E2 and progesterone levels as causative we have to show a maximum association between E2 and progesterone levels and reproductive outcomes. Nevertheless, in the current study, we did not find a strong association between serum progesterone and primary outcomes measures.

Improving receptivity in patients with high E2 levels is easy with suspension of ET to the next cycle. However, slightly increased miscarriage rates with FET further supports the possibility of a receptivity defect secondary to high E2 levels. Concordantly, infertile women with hyperandrogenism have low HOXA-10 and β3-integrin expression, which suggests high androgens may have a detrimental impact on the endometrium (22, 23). An increase in circulating androgens might antagonize the expression of estrogen-dependent receptivity genes. Therefore, we strongly propose that the decline in implantation rates in FET cycles is not exclusively due to defective follicle development but also the result of failed receptivity secondary to high circulating E2 levels.

As opposed to our results, some studies reported that high serum E2 levels were not detrimental to embryo implantation. For this reason, one may believe that an increase in E2 levels does not significantly impair the endometrial micro-environment. Conception despite high or low serum E2 levels suggests that receptivity of the endometrium was not strictly related to serum estradiol levels. It should be remembered that good quality embryos coming from fresh or FET cycles may come through an E2-associated implantation defect. Finally, before recommending the routine use of frozen ET for women with implantation failure who have high serum E2 or progesterone levels, we have to find answers to queries such as the timing of FET cycles and cut-off values of high serum E2 and progesterone on the day of hCG.

## Figures and Tables

**Table 1:**
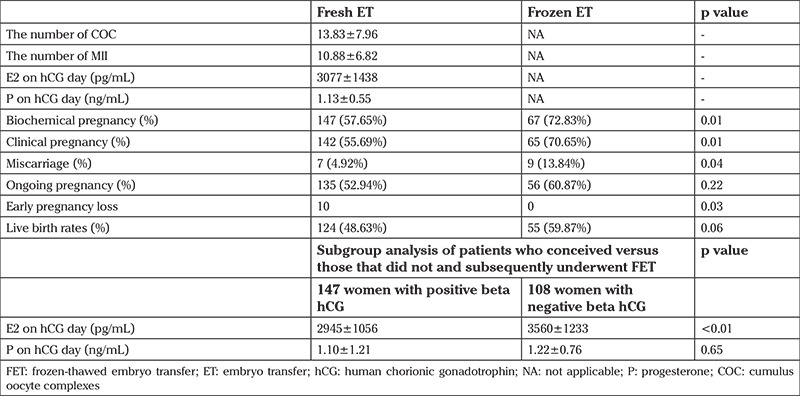
Clinical characteristics and sub-group analysis of fersh versus frozen-thawed embryo transfer cycles
